# Implementation of an interprofessional team-based learning program involving seven undergraduate health and social care programs from two universities, and students’ evaluation of their readiness for interprofessional learning

**DOI:** 10.1186/s12909-017-1046-5

**Published:** 2017-11-21

**Authors:** Lap Ki Chan, Fraide Ganotice, Frances Kam Yuet Wong, Chak Sing Lau, Susan M. Bridges, Celia Hoi Yan Chan, Namkiu Chan, Phoebe Wing Lam Chan, Hai Yong Chen, Julie Yun Chen, Jody Kwok Pui Chu, Charlene C. Ho, Jacqueline Mei Chi Ho, Tai Pong Lam, Veronica Suk Fun Lam, Qingyun Li, Jian Gang Shen, Julian Alexander Tanner, Winnie Wan Yee Tso, Arkers Kwan Ching Wong, Gordon Tin Chun Wong, Janet Yuen Ha Wong, Nai Sum Wong, Alan Worsley, Lei King Yu, Tin Pui Yum

**Affiliations:** 1 The University of Hong Kong, Hong Kong, People’s Republic of China; 2 The Hong Kong Polytechnic University, Hong Kong, People’s Republic of China; 3 School of Biomedical Sciences, Bau Institute of Medical and Health Sciences Education, Li Ka Shing Faculty of Medicine, The University of Hong Kong, Pok Fu Lam Hong Kong, People’s Republic of China

**Keywords:** Collaborative practice, Interprofessional education, Adult learning principles, Evaluation

## Abstract

**Background:**

Interprofessional learning is gaining momentum in revolutionizing healthcare education. During the academic year 2015/16, seven undergraduate-entry health and social care programs from two universities in Hong Kong took part in an interprofessional education program. Based on considerations such as the large number of students involved and the need to incorporate adult learning principles, team-based learning was adopted as the pedagogy for the program, which was therefore called the interprofessional team-based learning program (IPTBL). The authors describe the development and implementation of the IPTBL program and evaluate the effectiveness of the program implementation.

**Methods:**

Eight hundred and one students, who are predominantly Chinese, participated in the IPTBL. The quantitative design (a pretest-posttest experimental design) was utilized to examine the students’ gains on their readiness to engage in interprofessional education (IPE).

**Results:**

Three instructional units (IUs) were implemented, each around a clinical area which could engage students from complementary health and social care disciplines. Each IU followed a team-based learning (TBL) process: pre-class study, individual readiness assurance test, team readiness assurance test, appeal, feedback, and application exercise. An electronic platform was developed and was progressively introduced in the three IUs. The students’ self-perceived attainment of the IPE learning outcomes was high. Across all four subscales of RIPLS, there was significant improvement in student’s readiness to engage in interprofessional learning after the IPTBL. A number of challenges were identified: significant time involvement of the teachers, difficulty in matching students from different programs, difficulty in making IPTBL count towards a summative assessment score, difficulty in developing the LAMS platform, logistics difficulty in managing paper TBL, and inappropriateness of the venue.

**Conclusions:**

Despite some challenges in developing and implementing the IPTBL program, our experience showed that TBL is a viable pedagogy to be used in interprofessional education involving hundreds of students. The significant improvement in all four subscales of RIPLS showed the effects of the IPTBL program in preparing students for collaborative practice. Factors that contributed to the success of the use of TBL for IPE are discussed.

**Electronic supplementary material:**

The online version of this article (10.1186/s12909-017-1046-5) contains supplementary material, which is available to authorized users.

## Background

In the report titled “Framework for Action on Interprofessional Education & Collaborative Practice”, the World Health Organization stated that “interprofessional education and collaborative practice can play a significant role in mitigating many of the challenges faced by health systems around the world” [1]. An integrated health system can lead to improved patient satisfaction, patient acceptance of care and health outcomes, a more appropriate referral pattern, greater continuity and coordination of care, and collaborative decision making [2]. Integrated health care also reduces the effects of negative workplace interactions [3, 4].

However, healthcare students are traditionally educated in “silos” or within the confines of their discipline throughout their academic program, with little opportunity to learn with students from other disciplines. Hence, they may not know what practitioners in other professions know, think, or feel. Stereotypes about other health professions may form, which may create future obstacles in delivering effective and holistic patient-centered care. Learning in disciplinary silos does not prepare the students for collaborative practice [5]. Despite the importance of interprofessional education (IPE), health and social care programs have been slow to adopt IPE, because of difficulties such as scheduling, learner-level matching, long preparation time, financial support, and staff support [6].

For those schools that adopt IPE, there is significant diversity in educational strategies in implementing IPE. In a review by Abu-Rish et al., [6], the authors reported that strategies for IPE include small group discussion, patient case analysis, large group lecture, clinical teaching, direct patient interaction, and reflective exercises. None of the articles reviewed by Abu-Rish et al. [6] used team-based learning (TBL) as the strategy, despite the fact that TBL is specifically designed to encourage teamwork [7, 8]. The suitability of TBL for IPE was only recently explored in a graduate medical education setting [9]. Ohtsuki and Matsui [10] gave a brief report on using TBL for IPE, but a full evaluation of an approach bringing together TBL and IPE with details on implementation, resources, and content materials has not been previously reported. Similarly, the work of Nelson et al. [11] provides a review of 17 studies conducted on interprofessional team training focusing on students at prelicensure level. However, owing to the great variety of methodologies (e.g., different teaching methods and assessment measures), they found little evidence to demonstrate the best way to implement team training. Their review mentioned that many studies focused on just one profession or on teams with limited disciplinary diversity (consisting mainly of medical and nursing students), which may not realistically reflect the diverse disciplinary composition of professional health-care teams.

In 2015, The University of Hong Kong (HKU) collaborated with The Hong Kong Polytechnic University (PolyU) to establish an interprofessional education program, using team-based learning as the pedagogy. The program is therefore called the interprofessional team-based learning program or IPTBL. From HKU, six undergraduate-entry health and social care programs participated: biomedical sciences, Chinese medicine, medicine, nursing, pharmacy, and social work. From PolyU, another six undergraduate-entry health and social care programs took part: medical laboratory sciences, nursing, occupational therapy, physiotherapy, radiography, and social work. In the first year (2016) of the implementation of the IPTBL program, which this paper describes, seven of the twelve programs took part (the six HKU programs and the nursing program from PolyU). The 2016 program consisted of three instructional units (IUs), each of which lasted for about four hours on a Saturday. The number of programs that took part in these IU ranged from three to six (Table 1).

**Table 1:** The number of students from different programmes participating in the three instructional units of the IPTBL programme

	*n*
Instructional Unit: Anticoagulation Therapy	
HKU Biomedical Sciences (year 4 of 4)	15
HKU Chinese Medicine (year 3 of 6)	24
HKU MBBS (year 4 of 6)	213
HKU Nursing (year 2 of 5)	192
PolyU Nursing (year 3 of 5)	40
HKU Pharmacy (year 4 of 4)	25
Total	**509**
Instructional Unit: Multiple Drugs and Complementary Therapies	
HKU Biomedical Sciences (year 4 of 4)	15
HKU Chinese Medicine (year 3 of 6)	24
HKU MBBS (year 4 of 6)	213
HKU Nursing (year 4 of 5)	206
HKU Pharmacy (year 4 of 4)	25
Total	**483**
Instructional Unit: Developmental Delay	
HKU MBBS (year 4 of 6)	213
PolyU Nursing (year 3 of 5)	81
HKU Social Work (year 4 of 4)	46
Total	**340**

This study describes the development and implementation of IPTBL, an IPE program using TBL as a pedagogy in the context of the medical education in Hong Kong. We explore the suitability of TBL as a pedagogy for a large number of students from a diverse spectrum of health and social care undergraduate-entry programs. Finally, we provide the results of an evaluation on the effectiveness of the program.

This study contributes to the IPE and TBL literature in various ways. First, it explores how TBL can be used to implement IPE involving two universities in an Asian context. Second, it describes the problems encountered and how they are addressed. Finally, it provides the initial evaluation data on the effectiveness of the project. We hope that this paper will serve as an example for other scholars involved in large-scale IPE.

## Methods

### Design

A pretest-posttest design, based on the responses of the students in self-report measures, was utilized to examine the initial gains of students in the program. The IPTBL program served as the intervention.

### Participants

Student-participants of this study consisted of 801 undergraduate students (*Mean* age = 21.28 years, *SD* = 1.32 years) pursuing health care and social care programs from two universities in Hong Kong (HKU and PolyU). They were predominantly Chinese. There were 280 males (35%) and 521 females (65%). They came from seven programs: HKU Biomedical Sciences, HKU Chinese Medicine, HKU Medicine, HKU Nursing, PolyU Nursing, HKU Pharmacy, and HKU Social Work (Table 1). The following programs have students who attended two or three IUs: HKU Biomedical Sciences, HKU Chinese Medicine, HKU MBBS, PolyU Nursing, and HKU Pharmacy. The students from the other two programs took part in just one IU.

Most of the students entered these programs immediately after they completed their education at a local or overseas high school (undergraduate-entry), although a minority of students have taken courses towards or even completed a university degree before they joined these programs.

The lengths of the participating programs are different: nursing 5 years, Chinese medicine 6 years, medicine 6 years, and the other programs 4 years. The IPTBL program targeted students who were in the latter half of their respective programs, when they had already developed certain aspects of their professional identity and competency (Table 1). The exceptions were the second-year nursing students and the third-year Chinese medicine students from HKU, because the senior-year students and their teachers from these two programs were not available to join the IPTBL program.

English is the sole medium of instruction in all the health and social care professional programs involved in the IPTBL, except for the Chinese medicine program, in which English is still the medium of instruction in the biomedical courses, although Chinese is used in other courses directly related to Chinese medicine. The IPTBL program was conducted in English.

For three of the seven participating programs, student participation in the IPTBL program was voluntary and students’ scores did not contribute to the summative score of any course in these programs. However, in the other four programs, students’ scores in the IPTBL contributed towards a certain percentage of the summative grade of a course in the respective program. For example, the social work students’ scores in the third instructional unit (IU) (on developmental delay) contributed 5% to the summative grade of “Advanced Social Work Practice II”, a course that they were taking at that time. For the nursing students in that IU, their marks contributed 10% to the summative score of “Child & Adolescent Health Nursing”, a course that they were taking in their nursing program.

Student participation in the evaluation of the IPTBL program was entirely voluntary. Students were informed that their participation or not would not affect their learning opportunities or scores. Written consent was obtained if students decided to participate. They were assured that the data would be treated with confidentiality and anonymity.

To prepare students for the IPTBL program, the first and second authors met with the students in each of the participating programs to introduce the IPTBL program, the learning outcomes, and the significance of IPE. They also described the TBL process and what students needed to do to prepare for the program (i.e. they needed to study some pre-assigned materials before coming to the face-to-face sessions, to be described in further detail in the latter part of the paper).

### Measures

Students were invited to indicate their readiness for IPE on the Readiness for Interprofessional Learning Scale (RIPLS, [12], Additional file 1), using a 5-point Likert scale from 1 (strongly disagree) to 5 (strongly agree), just before and after an IU. A paired t-test was used to examine the pretest and posttest score differences on the four subscales of RIPLS. RIPLS is a 19-item self-reporting instrument for assessing students’ readiness in interprofessional learning with students from other professional disciplines. It is an instrument that has been validated and used in various samples including Chinese [13]. It is composed of four subscales: teamwork and collaboration (α=.91), negative professional identity (α=.79), positive professional identity (α=.85), and roles and responsibilities (α=.71). These Cronbach alphas are based on our confirmatory factor analysis reported in another study on the psychometric properties of RIPLS involving predominantly Chinese participants, and were found to be acceptable and sound [14].

Student rating is one of the necessary sources of evidence of teaching effectiveness [15]. Therefore, at the end of each IU, students were invited to complete a questionnaire on their self-perceived attainment of the seven program-level learning outcomes. The students who agreed to take part indicated their perceived extent of attainment of the learning outcomes on a 5-point Likert scale from 1 (minimal achievement) to 5 (maximal achievement). Statistical Package for Social Sciences (SPSS) version 23 was used for the entire analysis.

## Results and discussions

### Program development

In choosing the pedagogy for the IPE program between HKU and PolyU, a number of factors were considered:

- Interactivity: The pedagogy must provide opportunities for students from different health and social care professional programs to learn with, about, and from one another, by allowing students to communicate and work together or even peer teach one another during the learning process.
- The number of students: IPE typically involves a large number of students since two or more health and social care professional programs with large student enrollments are involved [16]. A pedagogy for IPE needs to cater for this large number of students, which also complicates space allocation and timetabling. Therefore, pedagogies like problem-based learning, which has been adopted by HKU since 1997 to promote active learning, may not be suitable because of the large number of students and hence the large number of facilitators and rooms needed.
- Adult learning principles: The pedagogy for IPE should provide clear goals or outcomes to the students, motivate them to learn, engage them in active learning, and provide ample reflective opportunities [17]. Students should also be given ample and timely feedback during their learning [18, 19].
- Learning outcomes: Traditional pedagogies like lectures can cater for a large number of students [20], but are not very useful in helping students to achieve the intended learning outcomes in IPE, which are typically above the basic “knowledge” level (fact remembering) in Bloom’s taxonomy [21].
- Authenticity: IPE tends to give positive outcomes if authentic clinical scenarios are used to stimulate learning, in order to simulate the actual clinical context in which the students will work after their graduation [16].

Upon consideration of all these factors, we decided to adopt TBL as the pedagogy for the IPE program between HKU and PolyU. TBL was originally developed in the business school environment in the 1990s, in response to the need for active learning in ever-increasing class sizes [22, 23]. It is a “learner-centered, instructor-directed strategy for small group active learning in large group educational settings” [23]. Very few teachers are needed to facilitate a TBL session, even when there is a large number of students. It is also believed that inherent characteristics of TBL fit well in interprofessional education, because TBL provides deliberate opportunities for students to develop collaborative competency and other interprofessional competencies including communication, interpersonal skills, and teamwork skills [24].

We also decided to structure the IPTBL around six clinical areas because of the rich opportunities they offer to engage students from complementary health and social care disciplines in interprofessional learning: anticoagulation therapy, depression, fracture, multiple drugs and complementary therapies, developmental delay, and cancer. One IPTBL instructional unit (IU) was planned for each of the six areas. On the first year of the implementation of the IPTBL program, three of the six IUs were implemented (anticoagulation therapy, multiple drugs and complementary therapies, and developmental delay), while on the second year the first three would be run again along with the other three. The plan is to run the full IPTBL program consisting of all six IUs annually thereafter.

The project received significant support from the institutional leadership who clearly saw the need for the development of IPE. Owing to the complementary nature of the 12 programs from the two universities, their collaboration was deemed to benefit the students in all the programs. The leaders of all the 12 programs nominated teachers from the respective programs to take part in these six IUs, based on the relevance of a discipline in an IU (for instance, the program leader of physiotherapy would nominate a teacher in the IU on fracture because physiotherapists play an important role in the management of patients with fracture) as well as the availability of teachers who would be interested in joining a particular IU. Therefore, all of these six IUs had different combinations of professional programs. The IUs with the largest number of participating programs were IU on fracture and the one on cancer (with eight programs in each), and the IU with the least number of programs was developmental delay (with three programs). The number of students in each IU was therefore also different. The three IUs with the smallest number of students were selected for the first year of implementation of the IPTBL program (i.e., anticoagulation therapy, multiple drugs and complementary therapies, and developmental delay) because of logistic, space, and IT considerations. Table 1 shows the number of students in each of the disciplines that took part in these three IUs. Although they had the least number of students, two of them still involved around 500 students coming from five and six programs respectively, and the smallest IU still involved 340 students coming from three programs.

### Funding

The IPTBL program received funding support of about 5 million Hong Kong dollars over 3 years, from The University Grants Committee of Hong Kong, under the UGC Funding Scheme for Teaching and Learning Related Initiatives in 2012–2015 Triennium. The funding was mainly used for hiring (1) a full-time program coordinator with a background in education to be responsible for organizing and evaluating the program for 3 years at HKU; (2) a full-time project coordinator for 3 years at PolyU; and (3) a full-time information technology officer for one and a half years, for developing the IPTBL electronic platform. The funding also supported the faculty development activities, such as the running of workshops on IPE and TBL, both of which are relatively new to many teachers in HKU and PolyU.

### Intended learning outcomes

The IPTBL program has seven IPE intended learning outcomes [1, 25]. At the end of the IPTBL program, students, irrespective of the professional programs they belonged to, were expected to be able to:

1. collaborate with students in other professions in solving clinical problems;
2. compare roles, responsibilities, and limitations of different professions;
3. communicate opinions to other professionals and listen respectfully to others’ opinions;
4. critically review personal skills to enhance relationship within a team;
5. recognize the need to work collaboratively in the best interest of patients;
6. recognize the stereotypical views of other professionals held by themselves and others; and
7. recognize that views held by other professionals are equally valid and important.

The achievement of these seven program-level outcomes was supported by the seven IU-level outcomes of each IU, which can be mapped one-to-one to the program level outcomes. For example, in the IU on developmental delay, one outcome is “collaborate with students in other professions in diagnosing and managing children with developmental delay”, which can be mapped directly to the program-level outcome of “collaborate with students in other professions in solving clinical problems”.

### Venue

The face-to-face sessions of the three IUs in the first year of implementation of the IPTBL program were held in three lecture theatres at the Li Ka Shing Faculty of Medicine, The University of Hong Kong. Each theatre could accommodate about 200 students. The lecture theatres were separated by thick dividers which could be lifted up to combine two or even three into one bigger theatre. The seats were fixed on a tiered floor and designed to have a clear view of the screen at the front. The seats were labelled before the face-to-face sessions so that when students were seated according to our seating plan, they would form pre-planned interprofessional teams. However, during team activities, students were encouraged to stand up and turn around to talk to their team members who might be in an adjacent row, or even move into the open space in the lecture theatre such as the stairs or the front stage.

### Team formation

Central to TBL for IPE is the formation of interprofessional teams. Each of these teams went through the TBL process described below, through which students learned with, about, and from one another across interprofessional boundaries. For example, in the IU on anticoagulation therapy, students from biomedical sciences (15), Chinese medicine (24), medicine (213), nursing (232), and pharmacy (25) were mixed to form interprofessional teams. However, the team size needed to be kept at about six students per team, in order to ensure that every student would take part in team discussion. A large number of teams were thus formed and there were not enough, for instance, pharmacy students to include one in every team. Therefore, every team had two to three medical students, two to three nursing students, but only one student from one of the following programs: the biomedical sciences, Chinese medicine, or pharmacy. The interprofessional composition was not the same across all the teams even in a single IU; e.g., some but not all teams included a pharmacy student. Different teams were formed for different IUs. The students did not know who their teammates would be and there was no team activity before the face-to-face in each IU.

However, on the day of the face-to-face session, some students were absent, thus disrupting the original team formation plan. Team reformation was then conducted by the following rules: (a) each team must have five to seven members; (b) a team with four members or less could join the nearest team with three or less members provided that the newly formed team had five to seven members, and had students from at least two disciplines.

In the first IU (Anticoagulation therapy), of the 509 students expected to attend, 475 came (93.32% attendance rate). In the second IU (Multiple drugs and complementary therapies), 362 of 482 attended (75.10% attendance rate). In the third IU (Developmental delay), 168 of 340 attended (49.41% attendance rate). A large number of medical students were absent from the second and third IUs because some of them needed to go to clinical learning sessions. The number of teams that were dissolved (their students went to other teams to form new teams) were: four in the first IU; 18 in the second; and 24 in the third.

### Team-based learning implementation

**Fig. 1:**
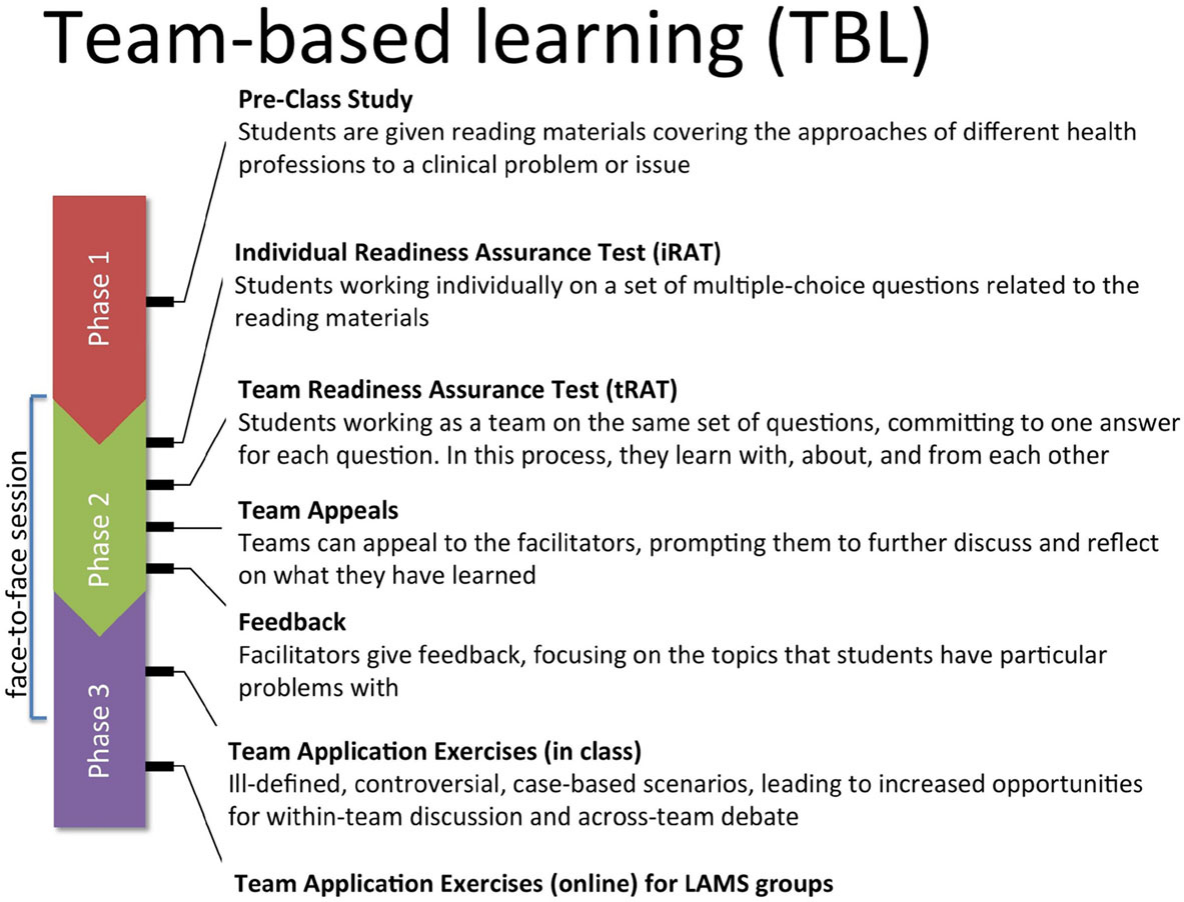
TBL steps in the IPTBL programme

Each of the three IUs implemented in the first year of the IPTBL program had one four-hour face-to-face session (Fig. 1), which was scheduled on a Saturday, because it was much easier logistically than trying to identify a weekday on which all the participating programs in an IU had no teaching and learning activities.

A new electronic platform had been developed for running the face-to-face session, though it was introduced only progressively (see below). Traditionally, TBL is done using printed materials through the various steps. However, technology can potentially facilitate the management of the complex but structured TBL process, especially with such a large number of participants. Whilst there is a considerable amount of literature highlighting the benefits of TBL in paper format [26], the literature is silent on the learning experiences and students’ gains when TBL is implemented and enriched with technology. The Lee Kong Chian School of Medicine of Nanyang Technological University has adopted the Learning Activity Management System or LAMS (developed by LAMS Foundation, LAMS International, and the Macquarie E-learning Centre of Excellence) for running their TBL sessions (Preman Rajalingam, personal communication). It is a system for creating, managing, and implementing sequences of learning activities which can be customized to suit specific requirements. After comparing different platforms, we adopted LAMS to develop a new electronic platform for facilitating the IPTBL program, which took about 1 year. Students brought their own mobile devices for the face-to-face session, and accessed the online LAMS platform wirelessly. They received the TBL materials and submitted their answers on the LAMS platform, and no printed materials were needed**.** Teachers controlled the release of the materials for the different TBL steps via the LAMS platform, which also provided the teachers with an overview of the progress of all students in real time.

Even though we had conducted extensive testing on the new LAMS platform, it was introduced through a deliberately cautious and progressive process with careful monitoring of the student usage, as well as network and server functions. Therefore, during the face-to-face session of the first of the three IUs (on anticoagulation therapy), the students were divided into two groups, which went through the IU simultaneously in two different venues (Table 2). The smaller group consisted of about one third of the 500 students and only the team leaders accessed the LAMS platform using their own mobile devices, while the larger group used paper. The assignment of the smaller group to LAMS aimed to test the LAMS platform, server, and network with a small number of users. For the second IU (on multiple drugs and complementary therapies), students were similarly divided into a larger and a smaller group, but this time, the team leaders of the larger group used LAMS, while the smaller group used paper. This is to challenge the LAMS platform, server and network with a larger user load. Because of the favorable usage data in the first and second IUs, all students, including team leaders and members in the third IU (on developmental delay) used LAMS (Table 2).

**Table 2:** Implementation characteristics of the three instructional units in the first year of implementation of the IPTBL programme

	First IU: Anticoagulation therapy		Second IU: Multiple drugs and complementary therapies		Third IU: Developmental delay
TBL by paper or LAMS	Paper	LAMS	Paper	LAMS	LAMS
Student distribution	2/3 of students	1/3 of students	1/3 of students	2/3 of students	All students
iRAT	On paper	On paper	On paper	On paper	On LAMS, by all students
tRAT	On paper	On LAMS, by team leaders	On paper	On LAMS, by team leaders	On LAMS, by team leaders
Face-to-face Application exercise	On paper	On LAMS, by team leaders	On paper	On LAMS, by team leaders	On LAMS, by team leaders
Online application exercise	No	Yes	No	Yes	Yes
Teachers	5 (one from each participating programme)	5 (one from each participating programme)	5 (one from each participating programme)	5 (one from each participating programme)	3 (one from each participating programme)

#### Pre-class study

Before students attended a face-to-face session (Fig. 1), they were given pre-class study materials such as book chapters, journal papers or even video clips, which they were expected to read or watch. Each of the disciplines involved in an IU contributed one study material. In choosing the materials, primary consideration was given to their relevance and appropriateness to the clinical case scenarios developed for the IPTBL program. The students were provided with the links to these materials 3 weeks before the face-to-face session through emails and could access these materials through their respective university libraries. Requiring the students to prepare before the face-to-face session encourages them to be responsible for their role in an interprofessional team.

#### Readiness assurance

When students came to the face-to-face session (Fig. 1), they sat in pre-assigned seats, so that they were surrounded by the members of their pre-planned interprofessional teams. But before they started any team activities, they needed to take a 10-minute test on the pre-class materials individually (called the individual readiness assurance test or iRAT), consisting only of multiple-choice questions selected from a pool created by the teachers involved in the IU. Approximately the same number of questions were selected from each participating discipline. In both the paper and the LAMS groups in the first and second IUs, the iRAT questions were printed on paper and the students indicated their answers on paper answer sheets that were collected immediately after the iRAT (Table 2). Only in the third IU, with the full adoption of LAMS, were the iRAT questions shown on the screen of the students’ mobile devices and students able to choose their answers by clicking on the screen.

After the iRAT, students formed pre-planned teams and would get to know one another through an ice-breaking game, which took about 15 min. After the ice-breaking game, students worked in teams and spent 15 min to discuss with their teammates the same test they had just taken individually. The team arrived at an answer for each multiple-choice question. The test was thus called the team readiness assurance test (tRAT). Taking the tRAT is one of the TBL activities which can stimulate interprofessional communication through which students can learn with, about, and from one another. The readiness assurance process underscores students’ accountability for coming to class prepared, and working together as a team in arriving at an answer that represents the consensus of the team [24].

During tRAT, students were provided with immediate feedback on whether they had chosen the correct answers. For teams in the paper IPTBL group, students indicated their team answers on an IF-AT form (Immediate Feedback Assessment Technique, Epstein Educational Enterprises) by scratching away the opaque films covering the chosen answer option. If the team had chosen the correct answer, a star or other symbol would be uncovered, so that the team knew they got the correct answer. But if the team got an incorrect answer (i.e. no star or symbol was uncovered), the team members needed to discuss again, choose another answer, and repeat the process as needed until they got the correct answer, though the team would get progressively lower marks for a question in their second, third, and fourth attempt (each multiple-choice question has only four options). The students in a team calculated their total score on the tRAT before the forms were collected by the teachers, who later rechecked the calculations. For the LAMS groups, only the team leader of each team could submit the team answers via the LAMS platform, while the team members could access the questions and options in LAMS but could not submit answers (Table 2). The team leader, and thus the team members as well, got immediate on-screen feedback through a scratch-and-reveal animation on the LAMS platform, similar to the IF-AT form. The total tRAT score for each team was calculated by the LAMS system and was shown to the team after it has completed the tRAT.

After the tRAT, students could appeal if they disagreed with the suggested correct answer. Teachers also took this opportunity to provide additional feedback on concepts and areas that students might have misunderstood or found difficult (Fig. 1). The time this step took was variable because teachers needed to choose among all the appeals and then decide which ones to respond to during the face-to-face session. In the paper groups, teams wrote their appeals on a paper form provided by the teachers. To help teachers screen the large number of appeals, appeal forms of two different colors were provided, one for concerns on the clarity and writing issues of the question, and another for content issues. These appeals were collected and sorted, and then the teachers decided which ones to respond to. In the LAMS groups, team leaders typed their appeals on the LAMS platform. These appeals were then displayed on the teachers’ LAMS interface, sorted according to frequency and nature. This feature of LAMS helped teachers screen the appeals more efficiently.

#### Application exercise

An application exercise is central to TBL [7]. In each face-to-face session, student teams were provided with one clinical scenario and they needed to tackle five multiple-choice questions based on that scenario (Fig. 1). The questions were in the one-best-answer multiple-choice format. These questions were designed to stimulate interprofessional discussion and to help students achieve the IPE outcomes.

In the paper groups, the clinical scenario was printed on paper, while in the LAMS group, it was displayed on the LAMS platform. The questions for the clinical scenario were tackled one at a time simultaneously by all the teams. The students were not able to see the subsequent questions when tackling one. This could be easily controlled in the LAMS groups because the teachers had control over when a question could be accessed by the students. In the paper groups, it was done by printing the questions on pieces of paper with distinctly different colors (for example, question one was printed on yellow paper and the second question on white paper, etc). After answering a question by circling the answer on the question sheet, the team needed to put the sheet into an envelope and never pull it out again. Since the question sheets all had distinctly different colors, the teachers could still easily find out if a team pulled out an earlier sheet (after the correct answer had been revealed).

In both the paper and LAMS groups, after the team had submitted their answer to a question, all the teams needed to simultaneously raise one of the four provided cards corresponding to their selected answer; e.g., if a team chose A for a question, the team leader needed to raise the orange card (the A, B, C, and D cards have different colors). Since all teams raised their cards simultaneously, teams could not choose their answers based on the choices of the other teams. It also gave the teachers a visual impression of the spectrum of the team answers. Based on the spectrum of answers, the teachers then had the important task of facilitating interteam discussion, by inviting teams that had chosen different answers to defend their choices. During the discussion, the teachers also pointed out whenever appropriate the importance of working collaboratively and how it could improve the clinical outcomes of patient and the efficiency of the health and social care system.

In the paper groups, there were no more activities after the application exercise. But for the LAMS groups, students were provided with an additional clinical scenario in the LAMS platform, which they could continue to access for a week after the face-to-face session. During that week, the original team in the face-to-face session would engage in team discussion in an online forum in LAMS, to tackle five multiple-choice questions on the second clinical scenario. Once again, only the team leaders could submit the team answer.

### Faculty and materials development

On the first year of development of the IPTBL project, three workshops were organized to help teachers develop their competency in IPE and TBL. Experts in IPE and TBL were invited to run these workshops, but they were open to all teachers in both HKU and PolyU, although priority was given to those who had been nominated to take part in the IPTBL program. These workshops were from one to three full days long.

The teachers in each IU met about three times (about six hours in total) to develop the IU materials, which included identifying the preclass study materials, the multiple-choice questions for the iRAT and tRAT (based on the preclass study materials), two clinical scenarios (one for the face-to-face session and one for the online session) and multiple-choice questions for the application exercises based on the clinical scenarios. Apart from the face-to-face meetings, the development of the materials also benefited from the use of cloud computing, for teachers to simultaneously access and edit the materials being developed.

During the face-to-face session of an IU, be it a paper or LAMS group, there was at least one teacher present from each of the participating disciplines. Therefore, for the first IU (on anticoagulation therapy), there were five teachers in the paper group and another five teachers in the LAMS group. They were called the content experts. At the same time, for both the paper and the LAMS group, there was also another teacher (either the first or the second author of the paper) present whose function was to facilitate the smooth progress of the TBL steps. They were called the process experts. In the LAMS group, the information technology officer (ITO) was also present to help with the running of LAMS. A separate operational protocol was produced for each of the three roles (content expert, process expert, and ITO) which gives detailed guidance on what to do in each of the steps of a face-to-face session. In addition, the content experts received a checklist to guide them in preparing for the IPTBL program. The checklist is a synopsis of how TBL can be used to promote IPE.

### Evaluation

The students’ self-perceived attainment of the IPE learning outcomes was high. Among the seven outcomes, “*Recognize the stereotypical views of other health workers held by themselves and others*” received the lowest attainment (*M* = 3.60), while “*Communicate opinions to other professionals and listen respectfully to others’ opinions*” received the highest attainment (*M* = 3.82). Outcomes that relate to the need to collaborate with others, compare roles and responsibilities, and recognize the acceptability of views of others received 3.69 to 3.80 (Table 3).

**Table 3:** Students’ self-perceived attainment of learning outcomes on returned questionnaire from Instructional Units: Anticoagulation Therapy, Multiple Drugs and Complementary Therapies, Developmental Delay (*N* = 1005)

	Mean (SD)
1. I am able to collaborate with students in other professions in solving clinical problems.	3.75 (0.70)
2. I am able to compare roles, responsibilities, and limitations of different health professions	3.69 (0.73)
3. I am able to communicate opinions to other professionals and listen respectfully to others’ opinions.	3.82 (0.68)
4. I am able to critically review my personal skills to enhance relationship within a team.	3.72 (0.69)
5. I am able to recognize the need to work collaboratively in the best interest of patient.	3.80 (0.70)
6. I am able to recognize the stereotypical views of other health workers held by themselves and others.	3.60 (0.75)
7. I am able to recognize that views held by other health care workers are equally valid and important.	3.80 (0.68)

**Fig. 2:**
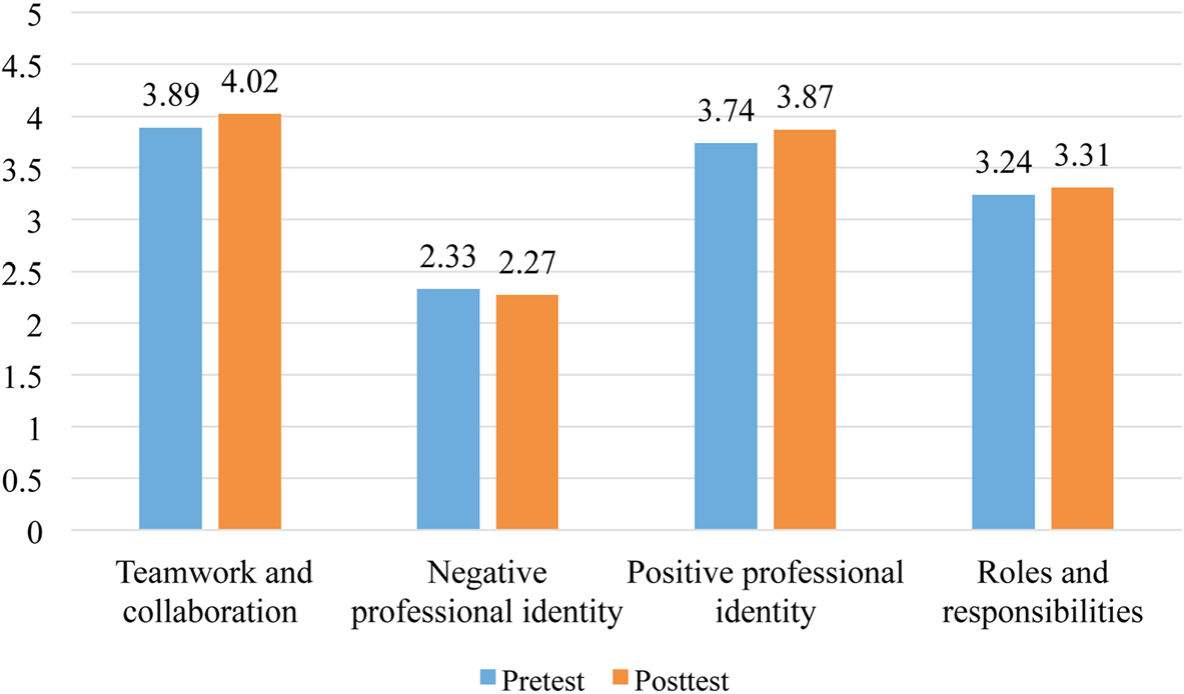
Pretest and posttest scores of students across the four subscales of Readiness for Interprofessional Learning Scale

Across all four subscales of RIPLS, there was significant improvement in students’ readiness to engage in interprofessional learning after the IPTBL (Fig. 2 and Table 4): teamwork and collaboration, negative professional identity, positive professional identity, and roles and responsibilities. These results are in the expected direction which can be interpreted as an effect of IPTBL program. Teamwork and collaboration, because of the largest mean differences, is the area where students demonstrated the biggest improvement. Although the change in students’ pretest-posttest scores was somewhat small, the differences were significant and meaningful for a one-time IPTBL intervention, especially in the context of large undergraduate-entry health and social care programs.

**Table 4:** Comparison of means of various subscales of Readiness for Interprofessional Learning Scale (RIPLS)

	Mean (SD)		*n*	*t*-value
	Pretest	Posttest		
1. Teamwork and collaboration**	3.89 (0.52)	4.02 (0.56)	676	7.687
2. Negative professional identity*	2.33 (0.79)	2.27 (0.86)	691	−2.178
3. Positive professional identity**	3.74 (0.57)	3.87 (0.60)	692	6.409
4. Roles and responsibilities**	3.24 (0.49)	3.31 (0.69)	695	3.503

*p < .05 **p < .01 for t-test of difference in means between pretest and posttest; scores on negative professional identity were not reversed coded to differentiate the subscale from positive professional identity

### Challenges and opportunities for improvement

While IPE is important for preparing health and social care students for future collaborative efforts, we experienced a number of difficulties in implementing it [6, 27]. In our initial implementation of the IPTBL program, the problems we encountered can be summarized into the following points:

#### Significant time involvement of the teachers

The most important resources needed for developing the IPTBL program are the teachers from the participating programs. Teachers spent a significant amount of time to go through the faculty development workshops on IPE and TBL, which were quite new to many of them. The numerous meetings also cost them extra time, though cloud computing saved them some. However, many teachers said it had been a very valuable experience for them, in learning to work with teachers from other disciplines. The teachers were learning with, from, and about one another, just as we expected our students to in the IPTBL program.

#### Difficulty in matching students from different programs

We targeted students in the senior years. We would like the students to have some differentiation into their professional disciplines so that they could learn with, about, and from one another. If we had done it in the first year of all the participating programs, the students would be too similar for them to learn about and from one another (though they can still learn with each other). However, this aim was not completely met since some students were from earlier years of two of the seven programs.

#### Difficulty in making IPTBL count towards a summative assessment score

In the first year of implementation of the IPTBL program, only four of the seven participating programs made IPTBL an integral part of a course in their programs, meaning that IPTBL contributed to part of the summative assessment score of a course in these programs. In the other three programs, the participation of their students was voluntary and had no contribution to summative assessment. This difference in the policy was believed to have affected students’ motivation and participation. In response to this, we made a strong appeal to all participating programs to integrate IPTBL into their curricula and make it count towards the summative assessment of a course in the program.

#### Developing the LAMS platform

The development of the LAMS platform is another big drain on resources in developing the IPTBL program. Although LAMS is a customizable modular platform for organizing teaching and learning activities, it still took a long time to customize it for team formation, appeals, etc. Moreover, the server and network had to be tested many times to ensure that they could support the large number of students in the face-to-face sessions.

#### Logistics difficulty in managing paper TBL with large number of students

The difficulty mainly concerns the management of the TBL process for a large number of students when paper is used for its various steps. Color coding of the papers for different TBL steps was done to facilitate the logistics and to prevent students from using the wrong papers (deliberately or accidentally). But this system was not perfect since it was still possible for students to change the answers to earlier questions after the correct ones had been revealed.

We developed LAMS to address many of the disadvantages of using paper for the IPTBL, but it still lacked some features which could have made a face-to-face session more efficient. For example, the existing version of LAMS does not allow the teachers to set a time limit on, say, iRAT, and it does not provide instant statistics on student performance. Such information would have been very useful to the teachers, for monitoring how the students were performing, what feedback to give, and what adjustments need to be made during the face-to-face sessions.

#### Inappropriateness of the venue

A lecture theatre was considered less appropriate for interaction and communication in the IPTBL program. While TBL can be done despite the space constraints, it is assumed that it is best done in a space where teams of students can gather in circles [28]. There is a link between physical arrangement and interaction patterns among team members. For example, members who sit in circles have an easier time communicating with each other, and seating patterns influence interaction patterns [29]. Some content experts expressed the need to explore other venues more conducive to interactions in TBL. For future implementation of the IPTBL program, venues with flat floors and mobile chairs have been identified.

### Limitations

Like any study, ours has a number of limitations. First, the students’ evaluation can be strengthened by means of triangulation and follow-up to examine the long term effect of the program to the students. Second, the one group pretest-posttest experimental design has a number of threats to internal validity (e.g., repeated testing). This can be addressed by employing a control group and administering the posttest after a reasonable period of time. Third, data on student evaluation presented here were collected immediately after the face-to-face session. More valid data on the effect of IPTBL can be collected in the future after the students start practicing their professions. How they value interprofessional collaboration in providing service to their patients may be a more authentic success indicator which can be construed as an effect of IPTBL. Lastly, we acknowledge the potential effects of students volunteering to fill out the evaluation questionnaire, although in our study, to encourage them to give objective feedback, we explained that their participation in filling out the questionnaires would not affect their grades and learning opportunities. We hope that future studies can remedy these concerns and design systems to administer IPTBL with attention to the limitations of the current study. These limitations notwithstanding, this study serves as an example in the development, implementation, and evaluation of an IPE program using TBL as the pedagogy, for a large number of students from undergraduate-entry health and social care programs.

## Conclusions

Our experience in using TBL to implement an IPE program for a large number of undergraduate health and social care programs (seven in our first year of implementation), involving about 300 to 500 students in each IU, indicated that TBL is a viable pedagogy for IPE. The significant improvement in all four subscales of RIPLS showed the effects of the IPTBL program on students in preparing them for collaborative practice. The interactions among the students, the scenario-based learning, the incorporation of adult learning principles and the need for relatively few facilitators probably all contributed to the success of the use of TBL for IPE.

We want to end this article by quoting a line from the report by VanKuikena et al., (p. 11), [30]: “While it has become clear that IPE is a preferred model for educating health professionals in the era of health care teams, we still have much to learn about how to best implement IPE…”. We hope that our initial experiences in implementing IPTBL is responsive to that call and will help other researchers and educators in developing IPE models in the future.

## Additional file

Additional file 1: Readiness for Interprofessional Learning Scale [12]. This scale was used to estimate the readiness of students from various programs to participate in interprofessional education. (DOC 85 kb)
