# Supplementary material for: Implementation of an interprofessional team-based learning program involving seven undergraduate health and social care programs from two universities, and students’ evaluation of their readiness for interprofessional learning
Source: BMC Med Educ. 2017 Nov 21;17:221. doi: 10.1186/s12909-017-1046-5 (PMC5697117; doi:10.1186/s12909-017-1046-5)
Supplement: Additional file 1: — Readiness for Interprofessional Learning Scale [12]. This scale was used to estimate the readiness of students from various programs to participate in interprofessional education. (DOC 85 kb) [file 12909_2017_1046_MOESM1_ESM.doc]

Table 5. Readiness for Interprofessional Learning Scale (RIPLS)

Directions: Please indicate how you feel about interprofessional learning by ticking the option button that best represents your assessment of yourself from 1 (strongly disagree) to 5 (strongly agree).

| **Teamwork and collaboration** |  |  |  |  |  |
| --- | --- | --- | --- | --- | --- |
| 1. Learning with other students will help me become a more effective member of a health care team. | 1 | 2 | 3 | 4 | 5 |
| 1. Patients would ultimately benefit if health-care students worked together to solve patient problems. | 1 | 2 | 3 | 4 | 5 |
| 1. Shared learning with other health-care students will increase my ability to understand clinical problems. | 1 | 2 | 3 | 4 | 5 |
| 1. Learning with health-care students before qualification would improve relationships after qualification. | 1 | 2 | 3 | 4 | 5 |
| 1. Communication skills should be learned with other health-care students. | 1 | 2 | 3 | 4 | 5 |
| 1. Shared learning will help me to think positively about other professionals. | 1 | 2 | 3 | 4 | 5 |
| 1. For small group learning to work, students need to trust and respect each other. | 1 | 2 | 3 | 4 | 5 |
| 1. Team-working skills are essential for all health care students to learn. | 1 | 2 | 3 | 4 | 5 |
| 1. Shared learning will help me to understand my own limitations. | 1 | 2 | 3 | 4 | 5 |
| **Negative Professional Identity** |  |  |  |  |  |
| 1. I **don’t** want to waste my time learning with other health-care students.* | 1 | 2 | 3 | 4 | 5 |
| 1. It is **not** necessary for undergraduate health-care students to learn together.* | 1 | 2 | 3 | 4 | 5 |
| 1. Clinical problem-solving skills can **only** be learned with students from my own department.* | 1 | 2 | 3 | 4 | 5 |
| **Positive Professional Identity** |  |  |  |  |  |
| 1. Shared learning with other health-care students will help me to communicate better with patients and other professionals. | 1 | 2 | 3 | 4 | 5 |
| 1. I would welcome the opportunity to work on small-group projects with other health-care students. | 1 | 2 | 3 | 4 | 5 |
| 1. Shared learning will help to clarify the nature of patient problems | 1 | 2 | 3 | 4 | 5 |
| 1. Shared learning before qualification will help me become a better team worker. | 1 | 2 | 3 | 4 | 5 |
| **Roles and Responsibilities** |  |  |  |  |  |
| 1. The function of nurses and therapists is mainly to provide support for doctors. | 1 | 2 | 3 | 4 | 5 |
| 1. I’m **not** sure what my professional role will be. | 1 | 2 | 3 | 4 | 5 |
| 1. I have to acquire much more knowledge and skills than other health-care students. | 1 | 2 | 3 | 4 | 5 |
